# Nirsevimab Effectiveness Against Medically Attended Respiratory Syncytial Virus Illness and Hospitalization Among Alaska Native Children — Yukon-Kuskokwim Delta Region, Alaska, October 2023–June 2024

**DOI:** 10.15585/mmwr.mm7345a1

**Published:** 2024-11-14

**Authors:** Brian Lefferts, Sara Bressler, James W. Keck, Christine Desnoyers, Ellen Hodges, Gerald January, Kristina Morris, Leslie Herrmann, Rosalyn Singleton, Sarah Aho, Julia Rogers, Katherine Newell, Elizabeth Ohlsen, Ruth Link-Gelles, Fatimah S. Dawood, Dana Bruden, Marc Fischer, Joseph Klejka, Heather M. Scobie

**Affiliations:** ^1^Yukon-Kuskokwim Health Corporation, Bethel, Alaska; ^2^Arctic Investigations Program, CDC; ^3^Alaska Native Tribal Heath Consortium, Anchorage, Alaska; ^4^Section of Epidemiology, State of Alaska Department of Health; ^5^Epidemic Intelligence Service, CDC; ^6^Career Epidemiology Field Officer Program, CDC; ^7^Coronavirus and Other Respiratory Viruses Division, National Center for Immunization and Respiratory Diseases, CDC.

**Summary**

**What is already known about this topic?**

To prevent severe respiratory syncytial virus (RSV) illness, nirsevimab is recommended for all infants aged <8 months (born during or entering their first RSV season) who are not protected through maternal vaccination and for children aged 8–19 months (entering their second season) who are at increased risk for severe RSV, including all American Indian and Alaska Native (AI/AN) children.

**What is added by this report?**

In Alaska’s Yukon-Kuskokwim Delta, nirsevimab was 89% effective in preventing RSV-associated hospitalization for infants in their first RSV season and 76% and 88% effective against medically attended illness for children in their first and second seasons, respectively.

**What are the implications for public health practice?**

Nirsevimab can prevent severe RSV illness among AI/AN infants and children entering their first and second RSV seasons.

## Abstract

Respiratory syncytial virus (RSV) is a leading cause of hospitalization among young children. Historically, American Indian and Alaska Native (AI/AN) children have experienced high rates of RSV-associated hospitalization. In August 2023, a preventive monoclonal antibody (nirsevimab) was recommended for all infants aged <8 months (born during or entering their first RSV season) and for children aged 8–19 months (entering their second RSV season) who have increased risk for severe RSV illness, including all AI/AN children. This evaluation in Alaska’s Yukon-Kuskokwim Delta region estimated nirsevimab effectiveness among AI/AN children in their first or second RSV seasons during 2023–2024. Among 472 children with medically attended acute respiratory illness (ARI), 48% overall had received nirsevimab ≥7 days earlier (median = 91 days before the ARI-related visit). For children in their first RSV season (292), nirsevimab effectiveness was 76% (95% CI = 42%–90%) against medically attended RSV illness and 89% (95% CI = 32%–98%) against RSV hospitalization. For children in their second RSV season (180), effectiveness against medically attended RSV illness was 88% (95% CI = 48%–97%). Nirsevimab is effective for preventing severe RSV illness among infants entering their first RSV season and children entering their second season with increased risk for severe RSV, including all AI/AN children.

## Introduction

Respiratory syncytial virus (RSV) is a leading cause of hospitalization among young children (*1*). Historically, American Indian and Alaska Native (AI/AN) children have experienced high rates of RSV-associated hospitalization, with threefold to sevenfold higher rates in Alaska’s Yukon-Kuskokwim Delta region than in other U.S. areas (*2*,*3*). In August 2023, CDC’s Advisory Committee on Immunization Practices (ACIP) recommended a long-acting monoclonal antibody (nirsevimab) for all infants aged <8 months born during or entering their first RSV season and for children aged 8–19 months entering their second season who are at increased risk for severe RSV illness, including all AI/AN children (*4*). In clinical trials among children in their first RSV season, nirsevimab efficacy was 79% for preventing medically attended RSV-associated lower respiratory tract infection and 81% for preventing RSV-associated hospitalization through 150 days after receipt (*4*). In September 2023, ACIP recommended that all infants be protected against severe RSV either through maternal RSV vaccination during pregnancy or infant receipt of nirsevimab; a majority of infants do not need protection from both products (*5*). This evaluation in Alaska’s Yukon-Kuskokwim Delta region provides the first real-world estimates of nirsevimab effectiveness among AI/AN children in their first and second RSV seasons.

## Methods

### Evaluation Site

The Yukon-Kuskokwim Delta region in southwestern Alaska includes approximately 27,000 persons (90% Alaska Native persons) living in the regional hub and 48 remote villages not connected by roads.* Yukon-Kuskokwim Health Corporation (YKHC), a tribal health organization, manages a regional hospital that performs RSV RNA testing and 46 village clinics that send swabs to the regional hospital for RNA testing. An estimated 1,591 children aged <20 months lived within the catchment area on October 1, 2023, the start of the RSV season^†^ (*4*). YKHC administered nirsevimab to 756 (48%) children aged <20 months during October 16, 2023–April 30, 2024.^§^

### Data Source and Inclusion Criteria

A deidentified database was developed that included demographic, clinical, laboratory testing, and immunization data from YKHC electronic health records and the state immunization information system.^¶^ Eligible children were aged <20 months on or born after October 1, 2023, living in the YKHC service area, and had a medically attended acute respiratory illness (ARI) visit at a YKHC facility during October 23, 2023–June 30, 2024. Medically attended ARI was defined as an outpatient visit or hospitalization with an ARI discharge diagnosis code** and RSV RNA testing of a respiratory specimen collected from 10 days before through 3 days after the visit. RSV tests were performed using Cepheid GeneXpert (multiplexed with SARS-CoV-2 and influenza A and B). After ARI visits within 30 days were combined, only the first occurring medically attended ARI was included for each child.^††^ Visits were excluded if the child 1) had received nirsevimab <7 days earlier, or had received >1 dose of nirsevimab on different dates, or 1 dose of palivizumab (a different preventive monoclonal antibody to prevent severe RSV); 2) had a mother who received RSV vaccine during pregnancy; 3) had received a negative RSV test result but had an RSV discharge code; or 4) was ineligible for nirsevimab.

### Data Analysis

Nirsevimab effectiveness against medically attended ARI associated with RSV infection was evaluated using a test-negative design. Case-patients were those who had received a positive RSV test result. Control patients had received a negative RSV test result. Children were stratified by their RSV season based on their age on October 1, 2023 (first season included those aged <8 months or born after October 1, and second season included those aged 8–19 months). Odds ratios and 95% CIs were estimated using multivariable logistic regression analysis comparing receipt of nirsevimab among case- and control patients. Regression models were adjusted for age in months at medical visit, sex, calendar month, residence community type, and presence of one or more high-risk underlying condition.^§§^ Effectiveness was calculated as (1 − adjusted odds ratio) × 100%. Sensitivity analyses were conducted excluding cases and controls in which SARS-CoV-2 or influenza virus was detected. Effectiveness was also determined by time since receipt and against hospitalization. Analyses were conducted using SAS (version 9.4; SAS Institute). This activity was reviewed by CDC, determined not to be research, and conducted consistent with applicable federal law and CDC policy.^¶¶^ YKHC and the Alaska Native Tribal Health Consortium approved this project.

## Results

### Characteristics of Children Included in the Evaluation

Overall, 472 children had medically attended ARI visits (14% hospitalization, 70% emergency department, and 16% outpatient clinic) meeting inclusion and exclusion criteria, including 68 (14%) patients with positive RSV test results and 404 (86%) patients with negative RSV test results (Table 1). The percentage of positive RSV test results peaked during November 2023–January 2024 (Figure). The median age at the medical visit was 9 months (range = 0–27 months); 292 (62%) children were in their first RSV season, and 180 (38%) were in their second season. Overall, 98% of children were AI/AN, 73% lived in villages outside the regional hub, and 16% had at least one underlying condition increasing the risk for severe RSV illness; these characteristics differed for children in their first and second RSV seasons, with higher percentages of children in their first RSV season living outside the regional hub and having a high-risk underlying condition.***

**TABLE 1:** Characteristics of eligible children in their first or second respiratory syncytial virus season who had medically attended acute respiratory illness, by respiratory syncytial virus test result and receipt of nirsevimab*^,^^†^ — Yukon-Kuskokwim Region, Alaska, October 23, 2023–June 30, 2024

Characteristic	Total no. (%)	RSV test result no. (column %)		Received nirsevimab no. (row %)	
		Positive	Negative	Yes	No
**All children, no. (row %)**	**472 (100)**	**68 (14)**	**404 (86)**	**227 (48)**	**245 (52)**
**Child's RSV season (age at start of season, Oct 1, 2023)**					
1st season (<8 mos)	**292 (62)**	39 (57)	253 (63)	161 (55)	131 (45)
2nd season (8–19 mos)	**180 (38)**	29 (43)	151 (37)	66 (37)	114 (63)
**Age group at medical visit, mos**					
0–5	**157 (33)**	22 (32)	135 (33)	92 (59)	65 (41)
6–11	**133 (28)**	16 (24)	117 (29)	64 (48)	69 (52)
12–17	**98 (21)**	18 (26)	80 (20)	44 (45)	54 (55)
18–23	**72 (15)**	10 (15)	62 (16)	24 (33)	48 (67)
24–27	**12 (3)**	2 (3)	10 (2)	3 (25)	9 (75)
**Sex**					
Female	**220 (47)**	30 (44)	190 (47)	107 (49)	113 (51)
Male	**252 (53)**	38 (56)	214 (53)	120 (48)	132 (52)
**Race**					
AI/AN	**464 (98)**	68 (100)	396 (98)	225 (48)	239 (52)
Other	**8 (2)**	0 (—)	8 (2)	2 (25)	6 (75)
**Residence community type**					
Hub town with regional hospital	**129 (27)**	28 (41)	101 (25)	45 (35)	84 (65)
Village	**343 (73)**	40 (59)	303 (75)	182 (53)	161 (47)
**High-risk underlying condition^§^**					
None	**396 (84)**	55 (81)	341 (84)	192 (48)	204 (52)
≥1	**76 (16)**	13 (19)	63 (16)	35 (46)	41 (54)
**Hospitalization**					
Yes	**64 (14)**	23 (34)	41 (10)	29 (45)	35 (55)
No	**408 (86)**	45 (66)	363 (90)	198 (49)	210 (51)

**Abbreviations:** AI/AN = American Indian or Alaska Native; ARI = acute respiratory illness; RSV = respiratory syncytial virus.

* Overall, 32 of 504 infants with ARI medical visits including receipt of RSV tests during the analysis period were excluded. Reasons for exclusion included being born to a mother who received RSV vaccination during pregnancy (14), receipt of nirsevimab <7 days before medical visit (nine), receipt of ≥1 dose of nirsevimab on different dates (five), ineligible for nirsevimab (non-Alaska Native in their second season without other risk factors) (two), receipt of palivizumab 124 days before the ARI visit (one), and receipt of a negative RSV test result but with an RSV discharge code (one).

^†^ Receipt of nirsevimab documented by state immunization information system or provider electronic health record.

**^§^** A subset of underlying medical conditions conferring higher risk for severe RSV illness was defined as chronic lung disease of prematurity (two); congenital heart disease (eight); immunocompromise (one); cystic fibrosis (zero); Down syndrome (two); neurologic, musculoskeletal conditions, or both (51); congenital airway abnormalities (one); reactive airway disease (17); or prematurity (nine); 10 children had one or more underlying conditions.

**FIGURE:**
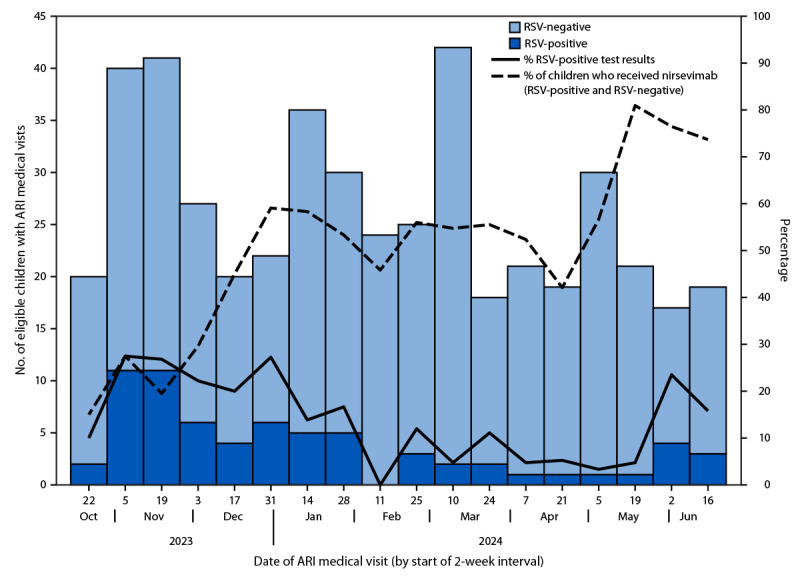
Trends in number of eligible children in their first or second respiratory syncytial virus season who had medically attended acute respiratory illness, by respiratory syncytial virus test result, test positivity, and receipt of nirsevimab* — Yukon-Kuskokwim Region, Alaska, October 23, 2023–June 30, 2024 **Abbreviations:** ARI = acute respiratory illness; RSV = respiratory syncytial virus. * Receipt of nirsevimab was calculated among eligible children with medically attended ARI medical visits and RSV-positive and RSV-negative test results. Receipt of nirsevimab was documented by state immunization information system or provider electronic health record.

### Receipt of Nirsevimab

Overall, 48% of all children had received nirsevimab ≥7 days before the ARI medical visit; this percentage was lower among children in their second RSV season (37%) and among those living in the regional hub (35%) than among children in their first season (55%) and those living in other villages (53%). Overall, among patients with positive RSV test results, 10 (15%) had received nirsevimab; 217 (54%) patients with negative RSV test results had received nirsevimab.

### Nirsevimab Effectiveness

Overall, nirsevimab effectiveness against medically attended RSV illness was 82% (Table 2). Among children in their first and second RSV seasons, effectiveness was 76% and 88%, respectively. Estimates were similar in sensitivity analyses excluding patients with specimens in which SARS-CoV-2 or influenza virus was detected (six with positive RSV test results and 86 with negative RSV test results) (Supplementary Table, https://stacks.cdc.gov/view/cdc/168888).

**TABLE 2:** Estimated nirsevimab effectiveness against medically attended respiratory syncytial virus illness and hospitalization, overall and by child’s respiratory syncytial virus season — Yukon-Kuskokwim Region, Alaska, October 23, 2023–June 30, 2024

Outcome/RSV season (age at start of season, Oct 1, 2023)	Nirsevimab dosage pattern	No. of patients	No. (row %)		Median no. of days since dose (IQR)	Adjusted effectiveness, % (95% CI)*
			RSV-negative	RSV-positive		
**Medically attended ARI**						
**Overall**	No nirsevimab doses (Ref)	245	187 (76)	58 (24)	NA	Ref
	Nirsevimab dose ≥7 days earlier	227	217 (96)	10 (4)	91 (45–141)	82 (62–91)
	Nirsevimab dose 7–89 days earlier	111	108 (97)	3 (3)	45 (27–68)	90 (68–97)
	Nirsevimab dose 90–179 days earlier	82	78 (95)	4 (5)	122 (103–142)	77 (31–92)
**1st season (<8 mos)**	No nirsevimab doses (Ref)	131	100 (76)	31 (24)	NA	Ref
	Nirsevimab dose ≥7 days earlier	161	153 (95)	8 (5)	82 (41–136)	76 (42–90)
**2nd season (8–19 mos)**	No nirsevimab doses	114	87 (76)	27 (24)	NA	Ref
	Nirsevimab dose ≥7 days earlier	66	64 (97)	2 (3)	106 (67–159)	88 (48–97)
**Hospitalization^†^**						
**Overall**	No nirsevimab doses (Ref)	35	15 (43)	20 (57)	NA	Ref
	Nirsevimab dose ≥7 days earlier	29	26 (90)	3 (10)	71 (36–118)	93 (64–99)
**1st season (<8 mos)**	No nirsevimab doses (Ref)	27	10 (37)	17 (63)	NA	Ref
	Nirsevimab dose ≥7 days earlier	22	19 (86)	3 (14)	73 (36–142)	89 (32–98)

**Abbreviations:** NA = not applicable; Ref = referent group; RSV = respiratory syncytial virus.

* Effectiveness was calculated as (1 − adjusted odds ratio) × 100%. Odds ratios were calculated using multivariable logistic regression, adjusted by age in months at medical visit (continuous), sex, calendar month of medical visit, residence community type, and presence of a high-risk underlying condition.

^†^ Effectiveness against hospitalization was not estimated for children in their second season because of small numbers (15).

Among children who received nirsevimab overall, the median interval from receipt to ARI medical visit was 91 days (range = 7–255 days) (Table 2) (Supplementary Figure, https://stacks.cdc.gov/view/cdc/168889). Effectiveness against medically attended RSV illness was 90% at 7–89 days after nirsevimab receipt and 77% at 90–179 days after receipt.

Overall, 64 children were hospitalized for ARI, including 23 patients with positive RSV test results, three of whom received nirsevimab. Nirsevimab effectiveness against RSV-associated hospitalization was 93% among children overall and 89% among children in their first RSV season, who accounted for 49 (77%) hospitalizations. Because of small numbers, effectiveness against hospitalization was not estimated for the 15 children who were in their second RSV season.

## Discussion

In this evaluation of 472 children with medically attended ARI in Alaska’s Yukon-Kuskokwim Delta region, nirsevimab was 89% effective against RSV hospitalization among children in their first season and 76% and 88% effective against medically attended RSV illness among children in their first and second RSV seasons, respectively. Consistent with previous studies among infants in their first RSV season (*6*–*8*), this evaluation documents nirsevimab effectiveness in an AI/AN population known to be at increased risk for severe RSV illness (*2*,*3*) and at a longer median interval from nirsevimab receipt (91 days) (*8*). Some evidence of waning overall effectiveness was observed (90% at 7–89 days and 77% at 90–179 days after receipt), but 95% CIs were wide and overlapped. These real-world estimates support current recommendations for nirsevimab to prevent severe RSV among infants in their first and second RSV seasons (*4*,*5*).

Compared with other U.S. data (*6*), a relatively high proportion of children aged <20 months in this evaluation (48%) received nirsevimab. In Alaska, all AI/AN children aged <8 months and aged 8–19 months in rural areas were prioritized to receive nirsevimab when shortages occurred during October 2023–January 2024.^†††^ Compared with an average of 56 RSV-associated hospitalizations among children aged <2 years in the region during nine previous RSV seasons (C Desnoyers, YKHC, and S Bressler, Arctic Investigations Program, CDC, personal communication, October 2024),^§§§^ 28 RSV-associated hospitalizations occurred during the 2023–24 season (including five RSV hospitalizations that were excluded in the evaluation^¶¶¶^), and three occurred in children who received nirsevimab ≥7 days earlier. An adequate, timely nirsevimab supply and increased coverage might further reduce RSV hospitalizations during the 2024–25 season (*9*).

### Limitations

The findings in this report are subject to at least five limitations. First, all RSV testing was clinician-directed, with inpatient and emergency visits accounting for the majority (84%) of included visits; possible exclusion of some children with milder illness could have affected estimated nirsevimab effectiveness. Second, low RSV incidence during the spring might have biased effectiveness estimates. Third, small numbers prevented estimation of effectiveness by time since receipt stratified by the child’s RSV season and against hospitalization among children in their second RSV season. Fourth, nirsevimab dosage**** was not ascertained, preventing effectiveness estimation by dosage. Finally, this evaluation was conducted predominantly among AI/AN children in one region of Alaska, and findings might not be generalizable.

### Implications for Public Health Practice

Nirsevimab was highly effective in preventing medically attended RSV illness and hospitalization among AI/AN children in Alaska’s Yukon-Kuskokwim Delta region during their first and second RSV seasons. These findings support current CDC recommendations for all infants in their first RSV season to either receive nirsevimab or be protected through maternal vaccination and for children entering their second season with increased risk for severe RSV illness, including all AI/AN children, to receive nirsevimab (*4*,*5*).

## Footnotes

*
https://data.census.gov/

† The first RSV case of the 2023–24 season in YKHC was identified on October 9, 2023.

§ Nirsevimab was first available in YKHC on October 16, 2023, and the period of administration was extended through April 30, 2024, in Alaska (https://health.alaska.gov/dph/Epi/Documents/phan/AKPHAN_20240319_RSVNextSteps.pdf). YKHC also provided RSV vaccine to 100 pregnant persons for the 2023–24 season.

¶ Current-season receipt of nirsevimab documented by state immunization information system or provider electronic health record.

**
https://knowledgerepository.syndromicsurveillance.org/cdc-broad-acute-respiratory-dd-v1

†† In scenarios in which multiple tests or ARI medical visits had discordant RSV test results (e.g., negative result followed by a positive result or positive result followed by a negative result), the visits were counted as RSV test–positive visits.

§§ High-risk underlying medical conditions were ascertained from the electronic health records, including chronic lung disease of prematurity, reactive airway disease, congenital heart disease, immunocompromise, cystic fibrosis, neuromuscular disease, congenital airway abnormalities that impair the ability to clear secretions, and prematurity.

¶¶ 45 C.F.R. part 46.102(l), 21 C.F.R. part 56; 42 U.S.C. Sect. 241(d); 5 U.S.C. Sect. 552a; 44 U.S.C. Sect. 3501 et seq.

*** Among children in their first season, 97% were AI/AN, 78% lived outside the regional hub, and 19% had underlying medical conditions. Among the children in their second season, 100% were AI/AN, 63% lived outside the regional hub, and 12% had underlying medical conditions. These differences in characteristics by the child’s RSV season were all statistically significant (p<0.05).

†††
https://health.alaska.gov/dph/Epi/Documents/phan/AKPHAN_20231025_NirsevimabSupplyTable.pdf

§§§ From regional surveillance data, this statistic is the 9-year average of RSV hospitalizations among children aged <2 years during the 2013–2020 and 2021–2023 seasons (excluding 2020–21 during the COVID-19 pandemic, which had no cases).

¶¶¶ Another three RSV hospitalizations in the YKHC service area that occurred in children were excluded from the analysis: one child received nirsevimab <7 days before the ARI visit, one received palivizimab 124 days earlier, and one was ineligible for nirsevimab because the child was not AI/AN and was aged 8–19 months; another two RSV hospitalizations were excluded because the child’s first ARI visit included in the evaluation period was >30 days earlier. None of these children received nirsevimab ≥7 days before the ARI visit.

**** Nirsevimab dosage is determined by the child’s age and weight. https://www.cdc.gov/vaccines/vpd/rsv/hcp/child.html
